# Comparative Mitogenomic Analysis Reveals Dynamics of Intron Within and Between *Tricholoma* Species and Phylogeny of Basidiomycota

**DOI:** 10.3389/fgene.2021.534871

**Published:** 2021-02-15

**Authors:** Wenli Huang, Huiyu Feng, Wenying Tu, Chuan Xiong, Xin Jin, Ping Li, Xu Wang, Qiang Li

**Affiliations:** ^1^Biotechnology and Nuclear Technology Research Institute, Sichuan Academy of Agricultural Sciences, Chengdu, China; ^2^Key Laboratory of Coarse Cereal Processing, Ministry of Agriculture and Rural Affairs, School of Food and Biological Engineering, Chengdu University, Chengdu, China; ^3^College of Life Sciences, Henan Agricultural University, Zhengzhou, China

**Keywords:** *Tricholoma*, mitochondrial genome, intron, gene rearrangement, evolution, phylogenetic analysis

## Abstract

The genus of *Tricholoma* is a group of important ectomycorrhizal fungi. The overlapping of morphological characteristics often leads to the confusion of *Tricholoma* species classification. In this study, the mitogenomes of five *Tricholoma* species were sequenced based on the next-generation sequencing technology, including *T. matsutake* SCYJ1, *T. bakamatsutake, T. terreum, T. flavovirens*, and *T. saponaceum*. These five mitogenomes were all composed of circular DNA molecules, with sizes ranging from 49,480 to 103,090 bp. Intergenic sequences were considered to be the main factor contributing to size variations of *Tricholoma* mitogenomes. Comparative mitogenomic analysis showed that the introns of the *Agaricales* mitogenome experienced frequent loss/gain events. In addition, potential gene transfer was detected between the mitochondrial and nuclear genomes of the five species of *Tricholoma*. Evolutionary analysis showed that the *rps3* gene of the *Tricholoma* species was under positive selection or relaxed selection in the evolutionary process. In addition, large-scale gene rearrangements were detected between some *Tricholoma* species. Phylogenetic analysis using the Bayesian inference and maximum likelihood methods based on a combined mitochondrial gene set yielded identical and well-supported tree topologies. This study promoted the understanding of the genetics, evolution, and phylogeny of the *Tricholoma* genus and related species.

## Introduction

As the “second genome” of eukaryotes, the mitochondrial genome plays an important role in eukaryotic growth and development, stress resistance, energy metabolism, aging, and death (Lang et al., 1999; Latorre-Pellicer et al., 2016). Mutations in the mitochondrial genome have been reported to affect disease development in animals (Gorman et al., 2015). The variation of the genome size, structure, gene content, gene arrangement, repeat sequences, and introns of the mitochondrial genome provides abundant information about the evolution and phylogeny of animals and fungi (Basse, 2010; Cameron, 2014; Li et al., 2020a). Fungi are one of the most diverse eukaryotic kingdoms on Earth (Hibbett et al., 2007). It is estimated that there are over 1.5 million fungal species. However, the mitochondrial genome of fungi is less understood than that of animals, especially Basidiomycetes. As of December 24, 2019, only 654 fungal mitochondrial genomes had been deposited in the National Center for Biotechnology Information (NCBI) database, of which <120 belong to Basidiomycetes. This shows that the mitochondrial genome of fungi is far from being fully understood. According to limited reports, the mitochondrial genome of fungi varies greatly in genome size, structure, gene arrangement, and intron classes, even between closely related species (Barr et al., 2005; Aguileta et al., 2014; Deng et al., 2018; Li et al., 2020b). Highly complex structures, abundant repetitive sequences, and large variations in gene content make it difficult to obtain complete fungal mitochondrial genome sequences, which limits our understanding of the fungal mitochondrial genome (Sandor et al., 2018; Liu et al., 2019; Wang et al., 2020a).

The genus *Tricholoma* is a group of important ectomycorrhizal fungi, which forms ectomycorrhizal relationships with trees from Fagaceae and Pinaceae (Li et al., 2018a). Through the ectomycorrhiza, plants have access to water and mineral elements and enhance their resistance to pathogens. In return, ectomycorrhizal fungi obtain a carbon source from host plants necessary for growth and fruiting (Vaario et al., 2019). The formation of this symbiotic relationship plays an important role in maintaining the forest ecosystem and promoting the carbon and nitrogen cycles in nature. It is reported that some nuclear genes of fungi play an important role in the adaptation to this symbiotic relationship, such as carbohydrate-degrading enzyme genes and transcription factors (Martin et al., 2008), while the mitochondrial genomes of ectomycorrhizal fungi are less understood.

Some species from the *Tricholoma* genus are valuable edible fungi, such as the pine mushroom *T. matsutake*, which are popular in Asia (Li et al., 2016a,b; Heilmann-Clausen et al., 2017). However, some other species from the *Tricholoma* genus, such as *T. bakamatsutake* and *T. sinoacerbum*, are not suitable for eating because of their pungent taste (Hosen et al., 2016). However, the two species have similar morphological characteristics, resulting in possible consumption by consumers (White et al., 2019). It is difficult to classify and identify *Tricholoma* species precisely because of their limited morphological features and the overlap of some morphological characteristics (Reschke et al., 2018; Endo et al., 2019). The mitochondrial genome is a powerful tool to study the phylogenetic relationships of species, and it has been widely used in the taxonomic study of animals (Boore, 1999). However, no mitochondrial genome has been used to study the phylogenetic relationships of the *Tricholoma* species.

In this study, the mitochondrial genomes of five species of *Tricholoma* were sequenced and assembled, including *T. matsutake* SCYJ1, *T. bakamatsutake, T. terreum, T. flavovirens*, and *T. saponaceum*. Comparing them with two published *T. matsutake* strains from Korea (Yoon et al., 2016) and Japan (LC385608), we revealed the features of *Tricholoma* mitogenomes and the variations or similarities in genome size, gene content, gene arrangement, and repeat sequences within and among *Tricholoma* species. The dynamic changes of introns and gene rearrangements in *Tricholoma* mitogenomes and other *Agaricales* mitogenomes were also revealed. In addition, the phylogenetic relationships of Basidiomycetes were analyzed based on a combined mitochondrial gene set. The mitogenomes of the five species of *Tricholoma* improve our understanding of the evolution, taxonomy, and genetics of this important ectomycorrhizal genus.

## Materials and Methods

### *De novo* Assembly and Annotation of Mitogenomes

The fruiting bodies of five *Tricholoma* species were collected from Sichuan, Yunnan, and Jilin provinces. The fruiting bodies were identified according to morphology and rRNA ITS sequences. The genomic DNA of the five *Tricholoma* species was extracted by a fungal DNA extraction kit (Omega Bio-Tek, Norcross, GA, USA) for sequencing library construction. We constructed sequencing libraries with the genomic DNA according to the instructions of NEBNext® Ultra™ II DNA Library Prep Kit (NEB, Beijing, China). Whole genomic sequencing was performed by the Illumina HiSeq 2500 Platform (Illumina, San Diego, CA, USA). The raw data obtained were first passed through a series of quality control steps, which included removing adapter reads using AdapterRemoval v2 (Schubert et al., 2016) and filtering low-quality sequences using ngsShoRT (Chen et al., 2014) with default parameters. The five mitogenomes were *de novo* assembled with the obtained clean data using the SPAdes 3.9 (Bankevich et al., 2012) with the k-mer of 17. Gaps among contigs were filled using the software MITObim V1.9 (Hahn et al., 2013). The MFannot tool (Valach et al., 2014) and MITOS (Bernt et al., 2013) were used to annotate the complete mitogenomes of the five *Tricholoma* species according to our previous described methods (Li et al., 2019a,b; Li et al., 2020c). OGDRAW (Lohse et al., 2007) was used to map the five mitogenomes of *Tricholoma*.

### Sequence Analysis

Base compositions of the five *Tricholoma* mitogenomes were analyzed using the DNASTAR Lasergene v7.1 (http://www.dnastar.com/). We assessed the strand asymmetry of the five mitogenomes according to the following formulas: AT skew = [A - T] / [A + T] and GC skew = [G - C] / [G + C] (Wang et al., 2017). MEGA v6.06 (Caspermeyer, 2016) was used to calculate genetic distances between each pair of the 15 core protein coding genes (PCGs), including *atp6, atp8, atp9, cob, cox1, cox2, cox3, nad1, nad2, nad3, nad4, nad4L, nad5, nad6*, and *rps3*, using the Kimura-2-parameter (K2P) model. DnaSP v6 (Rozas et al., 2017) was used to calculate the nonsynonymous substitution rate (*Ka*) and the synonymous substitution rate (*Ks*) for all of the 15 core PCGs in the *five Tricholoma* mitogenomes. We conducted the codon usage analysis using the Sequence Manipulation Suite (Stothard, 2000), based on the genetic code 4.

### Repetitive Elements Analysis

We conducted BLASTn searches of the five mitogenomes against themselves at an *E* value of <10^−10^ to determine whether there are intra-genomic duplications of large fragments and interspersed repeats in the five *Tricholoma* mitogenomes. Tandem Repeats Finder (Benson, 1999) was used to detect tandem repeats (>10 bp in length) in the five mitogenomes. Repeated sequences were also searched by REPuter (Kurtz et al., 2001) to identify forward (direct), reverse, complemented, and palindromic (reverse complemented) repeats in the five mitogenomes. We performed BLASTn searches of the five mitogenomes against their previously published nuclear genomes (Li et al., 2018b) to identify any gene segments that may have transferred between the mitochondrial and nuclear genomes of the five species (acc. Tmat, QMFF00000000.1; Tbak, QLOL00000000.1; Tter, QFEU00000000.1; Tfla, QLOK00000000.1; Tsap, QLOJ00000000.1).

### Intron Analysis

Introns of the core PCGs in the five *Tricholoma* mitogenomes and other *Agaricales* mitogenomes were classified into different position classes (Pcls) using the *Ganoderma calidophilum* mitogenome (Li et al., 2019c) as the reference according to the method described by Ferandon et al. (2013). The host genes of introns were aligned with *G. calidophilum* by Clustal W (Larkin et al., 2007). Each Pcl was constituted by introns inserted at the same position in the coding region of the PCGs. Introns belonging to the same Pcls usually contain high sequence similarities and are considered orthologous (Ferandon et al., 2010). Different Pcls usually show low sequence similarities and contain non-orthologous mobile genetic elements. The Pcls of core PCGs in *Agaricales* were named by number according to the insert position in the coding region of the host gene. The phylogenetic relationships of 27 *Agaricales* species were inferred based on the following phylogenetic methods.

### Phylogenetic Analysis

To investigate the phylogenetic status of the five *Tricholoma* species among the Basidiomycota phylum, we constructed a phylogenetic tree of 67 Basidiomycota species based on the combined mitochondrial gene set, which included 14 core PCGs. We first aligned single mitochondrial genes using MAFFT v7.037 (Katoh et al., 2019) and concatenated these alignments to a gene set using the SequenceMatrix v1.7.8 (Vaidya et al., 2011). Best-fit models of evolution and partitioning schemes for the gene set were determined according to PartitionFinder 2.1.1 (Lanfear et al., 2017). We used MrBayes v3.2.6 (Ronquist et al., 2012) to analyze the phylogenetic relationships of Basidiomycetes using a Bayesian inference (BI) method based on the combined gene set. Two independent runs with four chains (three heated and one cold) each were conducted simultaneously for 2 × 10^6^ generations. Each run was sampled every 100 generations. We assumed that stationarity had been reached when the estimated sample size (ESS) was greater than 100, and the potential scale reduction factor (PSRF) approached 1.0 (the closer the PSRF value is to 1, the better the convergence effect is). The first 25% samples were discarded as burn-in, and the remaining trees were used to calculate Bayesian posterior probabilities (BPP) in a 50% majority-rule consensus tree (Li et al., 2018c). The maximum likelihood (ML) method was also used to assess the phylogenetic relationships of 67 Basidiomycetes using RAxML v8.0.0 (Stamatakis, 2014) with the combined gene set. We assessed bootstrap values (BS) through an ultrafast bootstrap approach, with 10,000 replicates.

### Availability of Data

The five *Tricholoma* mitogenomes, including *T. matsutake* SCYJ1, *T. bakamatsutake, T. terreum, T. flavovirens*, and *T. saponaceum*, were submitted to GenBank under accession numbers MN873034, MN873035, MN873036, MN873037, and MN873038, respectively.

## Results

### Features of the Five *Tricholoma* Mitogenomes

**Figure 1:**
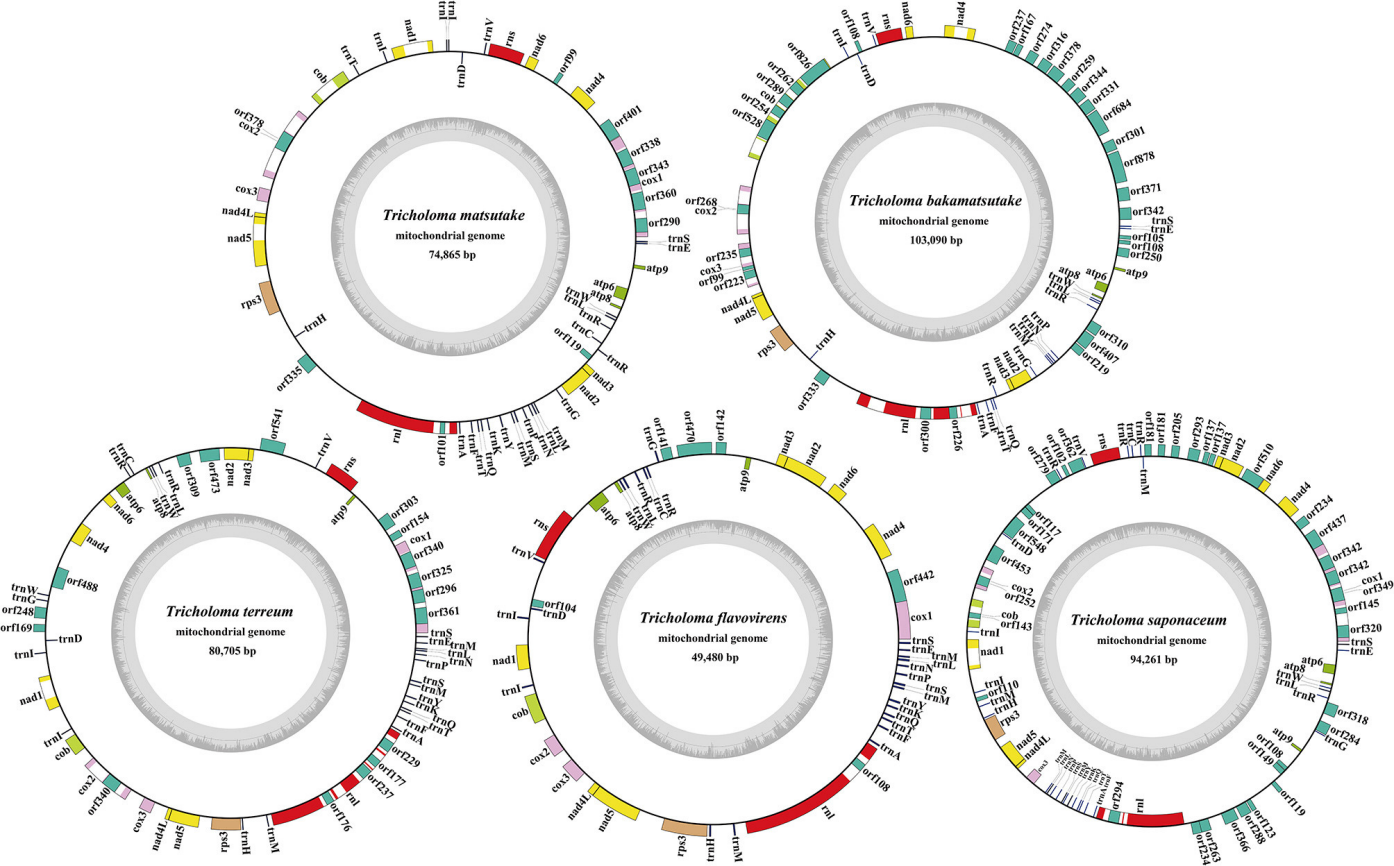
Circular maps of the mitochondrial genomes of five *Tricholoma* species. Genes are represented by different-colored blocks. Colored blocks outside each ring indicate that the genes are on the direct strand, while colored blocks within the ring indicate that the genes are located on the reverse strand.

The complete mitogenomes of the five *Tricholoma* species tested were all composed of circular DNA molecules, with sizes ranging from 49,480 to 103,090 bp (Figure 1). *T. bakamatsutake* contained the largest mitogenome among the five *Tricholoma* species, followed by *T. saponaceum, T. terreum*, and *T. matsutake*. The mitogenome of *T. flavovirens* was the smallest among the five *Tricholoma* species. The GC content of mitogenomes in the five *Tricholoma* species ranged from 20.57 to 23.03% (Supplementary Table 1). The GC content of the *T. saponaceum* mitogenome was the highest, while that of *T. matsutake* mitogenome was the lowest. The AT skews in mitogenomes of *T. terreum, T. matsutake*, and *T. bakamatsutake* were positive, while those in *T. flavovirens* and *T. saponaceum* were negative. GC skews of all five *Tricholoma* mitogenomes were positive. There were 19–42 non-intronic open-reading frames (ORFs) detected in the five *Tricholoma* species. Most *Tricholoma* species contained 14 core PCGs, except *T. bakamatsutake*, which did not contain the *nad1* gene. Non-conserved PCGs in the *Tricholoma* species mainly encoded DNA polymerase and proteins with unknown functions (Supplementary Table 2). A total of 57 introns were detected in the mitogenomes of five *Tricholoma* species, 80.70% of which contained intronic ORFs, which encoded LAGLIDADG homing endonuclease, GIY-YIG homing endonuclease, and putative reverse transcriptase. These introns were distributed in *cob, cox1, cox2, cox3, nad1, nad4*, and *nad5* genes. Most of these introns belonged to group I, and only two introns belonged to group II. All five *Tricholoma* mitogenomes contained two rRNA genes, namely the small subunit ribosomal RNA (*rns*) and the large subunit ribosomal RNA (*rnl*). The tRNA genes in the five *Tricholoma* mitogenomes ranged from 19 to 29.

### Overlapping Genes and Composition of Mitogenomes

We detected two overlapping genes in the mitogenomes of *T. matsutake, T. bakamatsutake, T. terreum*, and *T. flavovirens* (Supplementary Table 2). The four mitogenomes all contained a set of overlapping genes located across the neighboring genes *nad4L* and *nad5* (-1 bp). Four sets of overlapping genes were detected in the mitogenome of *T. saponaceum*, and the largest set of overlapping genes was located between *orf234* and *orf263* (-43 bp). A total of 34,053 bp, 35,257 bp, 34,505 bp, 20,344 bp, and 37,638 bp of intergenic sequences were detected in the mitogenome of *T. matsutake* SCYJ1, *T. bakamatsutake, T. terreum, T. flavovirens*, and *T. saponaceum*, respectively. The length of intergenic sequences ranged from 19 to 3,660 bp, and the longest intergenic sequence was located between *orf335* and *rnl* in the *T. matsutake* SCYJ1 mitogenome.

**Figure 2:**
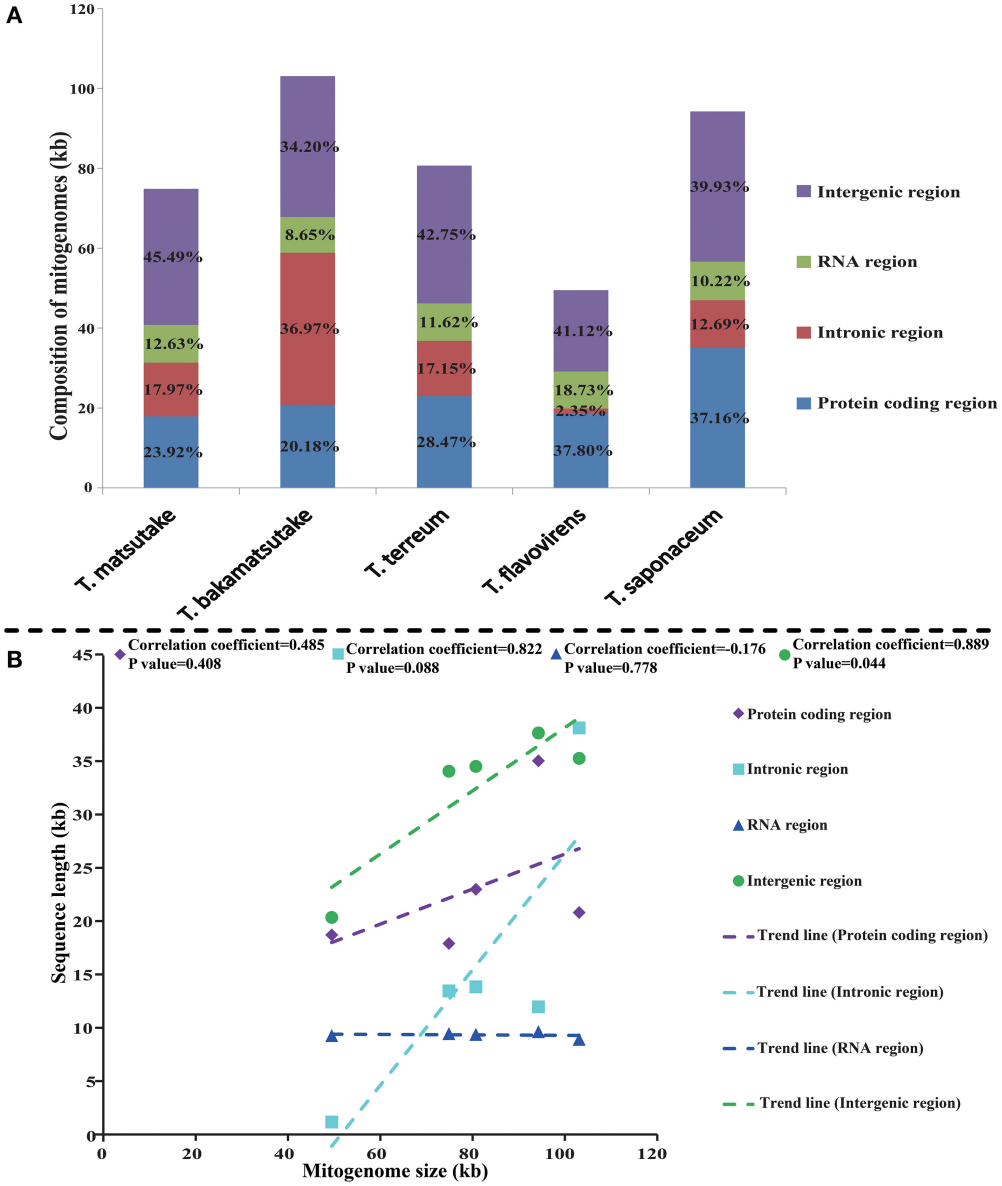
The protein-coding, intronic, intergenic, and RNA gene region proportions of the entire mitochondrial genomes of the five *Tricholoma* species **(A)** and pearson correlation analysis between mitogenome sizes and sequence lengths of 5 *Tricholoma* species **(B)**.

Among the five *Tricholoma* mitogenomes we tested, the protein-coding region accounts for the largest proportion, accounting for 40.21% of the complete mitogenomes on average, followed by the intergenic region, accounting for 28.68% of the five mitogenomes on average (Figure 2). Introns accounted for an average of 19.52% of the five *Tricholoma* mitogenomes. The proportion of the RNA region was the smallest, only accounting for 11.59% of the five mitogenomes. In the mitogenome of *T. bakamatsutake*, containing the largest mitogenome in the five *Tricholoma* species, introns accounted for the largest proportion (36.97%) of the entire mitogenome. *T. flavovirens*, which had the smallest mitogenome, contained only 2.35% of the intronic regions. Pearson correlation analysis indicated intergenic region was closely related to the size variation of mitogenome in *Tricholoma* (*P* < 0.05).

### Codon Usage Analysis

Most of the core PCGs in the five *Tricholoma* species used ATG as a start codon, while only the *cox1* genes of *T. matsutake* SCYJ1, *T. bakamatsutake* and *T. flavovirens* used GTG as a start codon (Supplementary Table 3). The *nad1, nad5*, and *nad6* genes of *T. terreum* used TAG as a stop codon, and TAG was also used as a stop codon of *nad2* in *T. matsutake* SCYJ1, *nad5* in *T. flavovirens*, and *nad6* in *T. saponaceum*. Other *Tricholoma* core PCGs used TAA as stop codons.

**Figure 3:**
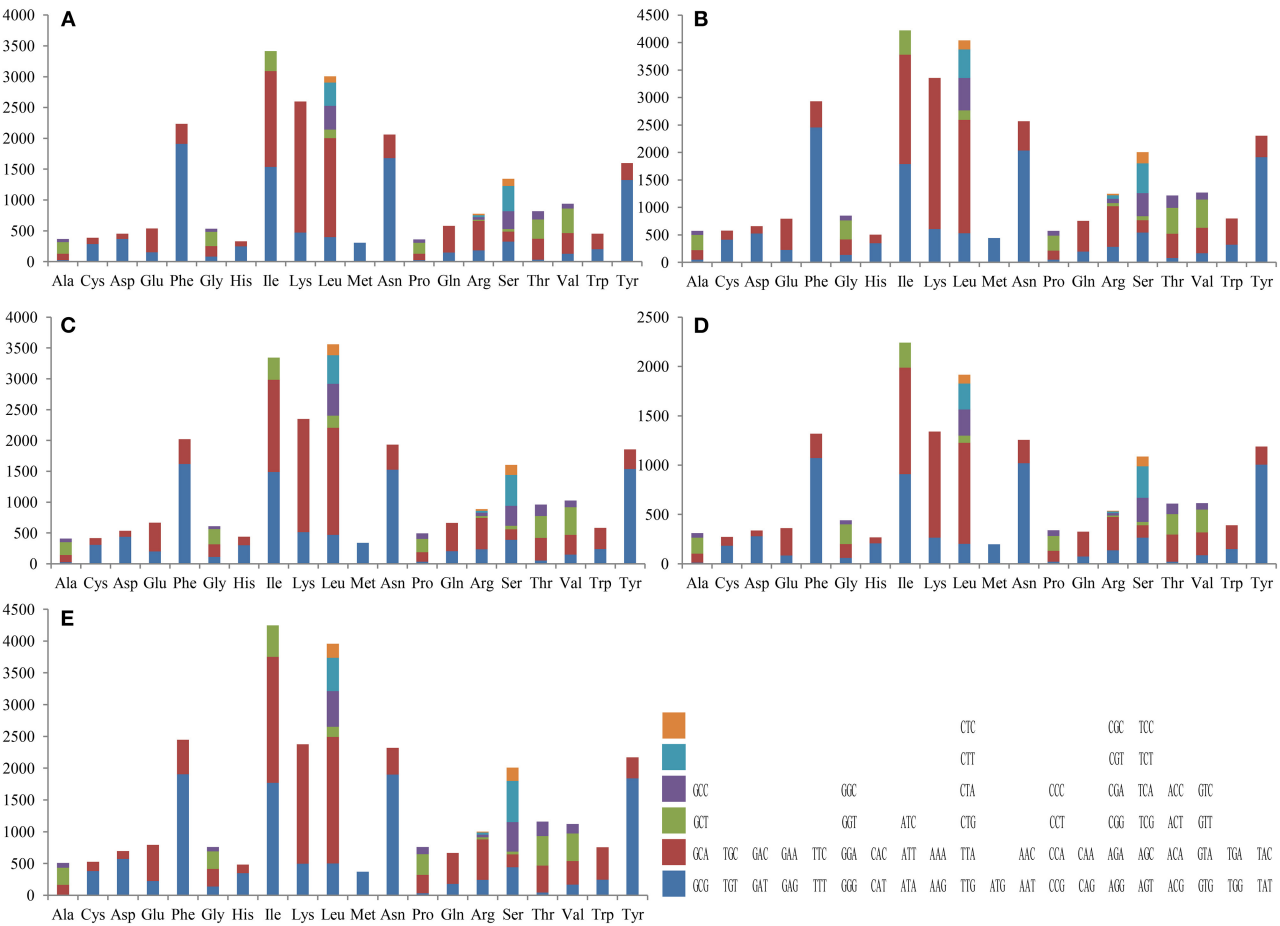
Codon usage in the mitochondrial genomes of five *Tricholoma* species. Frequency of codon usage is plotted on the y-axis: **(A)** *T. matsutake* SCYJ1; **(B)** *T. bakamatsutake*; **(C)** *T. terreum*; **(D)** *T. flavovirens*; and **(E)** *T. saponaceum*.

Codon usage analysis indicated that the most frequently used codons in the five mitogenomes were AAA (for lysine; Lys), TTT (for phenylalanine; Phe), AAT (for asparagine; Asn), TTA (for leucine; Leu), ATT (for isoleucine; Ile), and TAT (for tyrosine; Tyr) (Figure 3 and Supplementary Table 4). The frequent use of A and T in codons contributed to the high AT content in the *Tricholoma* mitogenomes (average: 77.70%).

### Repetitive Sequences Analysis

Comparing the whole mitogenomes of the five *Tricholoma* species with themselves *via* BLASTn searches, we identified nine repeat sequences in the mitogenome of *T. matsutake* SCYJ1, seven repeat sequences in *T. bakamatsutake*, 15 in *T. terreum*, 8 in *T. flavovirens*, and 31 in *T. saponaceum* (Supplementary Table 5). The length of repeat sequences in the five *Tricholoma* mitogenomes ranged from 28 to 1,139 bp, with pair-wise nucleotide similarities ranging from 76.21 to 100%. The largest repeats were detected in the protein-coding region of orf362 and intergenic region between *trnV* and orf362, as well as in the protein-coding region of orf366 and intergenic region between orf263 and orf366 in the *T. saponaceum* mitogenome. Repetitive sequences accounted for 1.10–6.86% of the whole mitogenomes of the five *Tricholoma* species. The mitogenome of *T. saponaceum* had the highest proportion of repeat sequences, followed by that of *T. terreum*, while *T. bakamatsutake* had the lowest proportion of repeat sequences.

A total of 154, 188, 67, 34, and 131 tandem repeats were detected in the mitogenomes of *T. matsutake* SCYJ1, *T. bakamatsutake, T. terreum, T. flavovirens*, and *T. saponaceum*, respectively (Supplementary Table 6). The longest tandem repeat sequence was observed in the mitogenome of *T. matsutake* SCYJ1, comprising of 247 bp. Most of the tandem repeats in the five *Tricholoma* mitogenomes were duplicated once or twice, with the highest replication number (115) in the *T. bakamatsutake* mitogenome. Tandem repeat sequences accounted for 3.20–12.78% of the five *Tricholoma* mitogenomes. Using REPuter, we identified 6 complemented, 17 forward, 5 palindromic, and 22 reverse repeats in the mitogenome of *T. matsutake* SCYJ1, accounting for 2.88% of the entire mitogenome (Supplementary Table 7). Repeats identified by REPuter accounted for 3.50, 3.44, 3.19, and 4.48% of the *T. bakamatsutake, T. terreum, T. flavovirens*, and *T. saponaceum* mitogenomes, respectively.

To detect if there were any gene segments that may had been transferred between the mitochondrial and nuclear genomes, we blasted the five mitogenomes against their nuclear genomes. A total of 75, 357, 104, 110, and 55 aligned fragments were detected in the mitogenome of *T. matsutake* SCYJ1, *T. bakamatsutake, T. terreum, T. flavovirens*, and *T. saponaceum*, respectively (Supplementary Table 8). The length of these aligned fragments ranged from 32 to 32,436 bp, with sequence similarities between 74.92 and 100%. The largest aligned fragment was located between the *orf548* and *rnl* genes, which encompassed the protein-coding regions of several conserved and non-conserved genes, as well as coding regions of several tRNA genes in the *T. saponaceum* mitogenome. The similarity of this large alignment is 99.87% between the nuclear sequence (acc. QLOJ01012658.1) and the sequence, with 17 mismatches and one gap (Supplementary Table 8). The large aligned sequences between respective mitochondrial and nuclear genomes of the five *Tricholoma* species indicated that genetic transfer between the mitochondrial and nuclear genomes may have occurred during the evolution of *Tricholoma* species.

### Variation, Genetic Distance, and Evolutionary Rates of Core Genes

**Figure 4:**
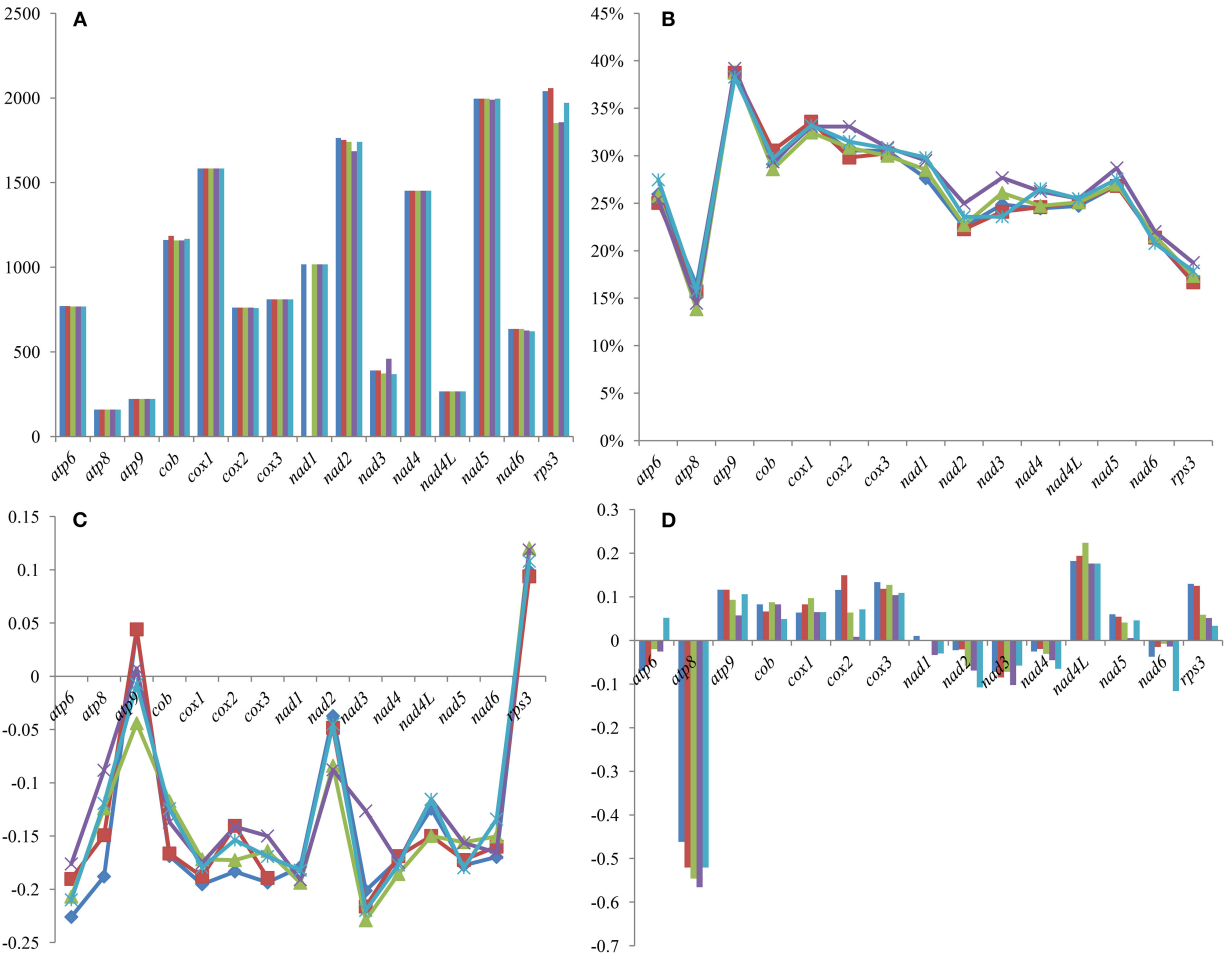
Variation in the length and base composition of 15 protein-coding genes (PCGs) between the five *Tricholoma* mitogenomes. **(A)** PCG length variation; **(B)** GC content of the PCGs; **(C)** AT skew; and **(D)** GC skew.

Among the 15 core PCGs we detected, the length of eight core PCGs varied between different *Tricholoma* species, including *atp6, cob, cox2, nad2, nad3, nad5, nad6*, and *rps3* genes (Figure 4). Among the genes with length variation, the *rps3* gene had the largest length variation, and no two *Tricholoma* species had an *rps3* gene of the same length. The GC content of *atp9* was the highest, and *atp8* was the lowest among the 15 core protein-coding genes. The GC content of all the core PCGs was different between different *Tricholoma* species, which indicated that there were frequent base variations in the core PCGs of *Tricholoma* species. Most core PCGs exhibited negative AT skews, except for *atp9* and *rps3*, which exhibited positive AT skews in one or all the five mitogenomes, respectively. The GC skews of core PCGs in the five mitogenomes were variable. The *atp8, nad2, nad3, nad4*, and *nad6* genes contained negative GC skews in the five *Tricholoma* species. However, GC skews in *atp9, cob, cox1, cox2, cox3, nad4L, nad5*, and *rps3* genes of the five mitogenomes were positive.

**Figure 5:**
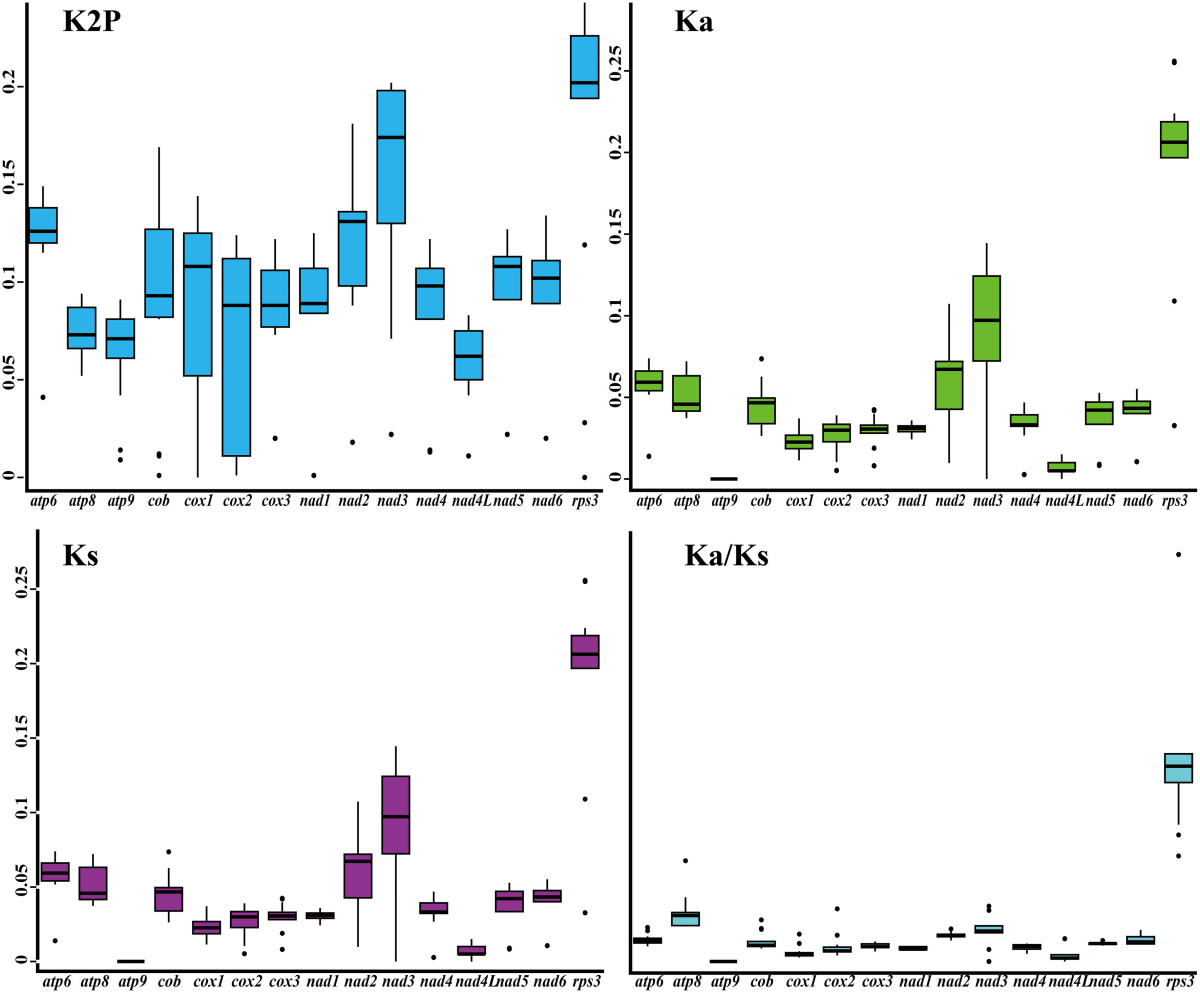
Genetic analysis of 15 protein-coding genes conserved in the five *Tricholoma* mitogenomes. K2P, the Kimura-2-parameter distance; Ka, the mean number of non-synonymous substitutions per non-synonymous site; Ks, the mean number of synonymous substitutions per synonymous site.

Among the 15 core PCGs detected, *rps3* gene had the largest K2P genetic distance between the five *Tricholoma* species on average, followed by the *nad3* gene (Figure 5). The *nad4L* gene had the smallest mean K2P genetic distance between the five *Tricholoma* species, indicating that this gene was highly conserved between *Tricholoma* species. The mean nonsynonymous substitution rate (*Ka*) of the *atp9* gene was the smallest, while that of the *rps3* gene was the largest in the *Tricholoma* species. The *atp9* gene had the smallest synonymous substitution rate (*Ks*) and the *rps3* gene had the largest *Ks* value among the 15 core PCGs. The *Ka/Ks* values of the 14 core PCGs used for energy metabolism were <1, indicating that these genes were subjected to purifying selection. However, the *rps3* gene contained a *Ka/Ks* value greater than one, indicating that this gene was subjected to positive selection or relaxed selection in some *Tricholoma* species.

### Intron Dynamics of PCGs in *Agaricales*

**Figure 6:**
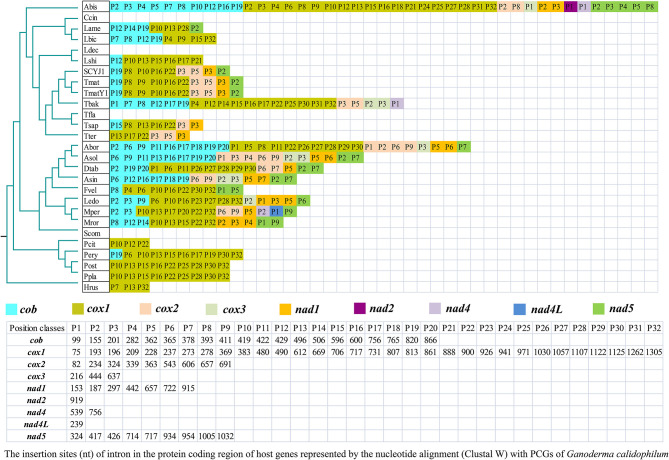
Position class **(**Pcl) information of core protein coding genes (PCGs) of the 27 *Agaricales* species. The phylogenetic positions of 27 *Agaricales* species were established using the Bayesian inference (BI) method and maximum likelihood (ML) method based on 15 concatenated mitochondrial core proteins. Species information is provided in Supplementary Table 9.

A total of 289 introns were detected in the core PCGs of 27 *Agaricales* species we tested. The number of introns in the core PCGs of each *Agaricales* species ranged from 0 to 41, indicating that the loss or gain of introns occurred frequently in the *Agaricales* species (Figure 6). Introns were found to be distributed in *cob, cox1, cox2, cox3, nad1, nad2, nad4, nad4L*, and *nad5* genes of *Agaricales*, and the dynamic change of introns promoted the large variation of mitogenome size in *Agaricales*. The *cox1* gene was the largest host gene of these introns, harboring 140 introns (48.44%), followed by the *cob* gene, which harbored 64 introns (22.15%). Only one intron was detected in the *nad2* and *nad4L* genes. According to the insertion site of introns in the protein-coding region of the host gene, we divide the introns into different position classes (Pcls). The introns from the same PCG belonging to the same Pcl were considered to be orthologous introns, which had high sequence similarity and usually contain orthologous intronic ORFs (Ferandon et al., 2010). Among the 27 *Agaricales* species tested, 84 Pcls were detected in core PCGs, with 32 in the *cox1* gene and 20 in the *cob* gene. Only one Pcl was detected in the *nad2* and *nad4L* genes. Pcls P10, P22, P16, P32, and P13 were widely distributed introns in the *cox1* gene, which was distributed in more than 10 out of the 27 *Agaricales* species. However, P2, P3, P5, P7, and P14 were only detected in one of the 27 *Agaricales* species, which were considered to be rare Pcls in *Agaricales*. These rare Pcls were also detected in distant species, such as *Austropuccinia psidii* (MN018834), *Heterobasidion irregular* (KF957635), and *Paxillus rubicundulus* (Li et al., 2020c), indicating that potential intron transfers might occur in the mitogenomes of *Agaricales* or the intron insertions were convergent in distant species. Pcl P19 was the most widely distributed Pcl in the *cob* gene, which was distributed in 12 of the 27 species, while Pcls P1, P4, and P5 were rare Pcls in the *cob* gene and only distributed in one of the 27 *Agaricales* species.

Among the 7 *Tricholoma* species tested, we found that the number and position class of introns varied significantly within or between species (Figure 6). *T. bakamatsutake* had the largest number of introns in the core PCGs among the seven *Tricholoma* species, while *T. flavovirens* did not contain any intron in core PCGs. Several Pcls, including P22 in the *cox1* gene, P3 in the *cox2* gene, and P3 in the *nad1* gene, were considered widely distributed Pcls in *Tricholoma* species. Interestingly, within the *T. matsutake* species, *T. matsutake* SCYJ1, which was collected from the Sichuan province, China, lost the Pcl P9 of the *cox1* gene compared with the *T. matsutake* species collected from Korea and Japan.

### Gene Arrangement and Phylogenetic Analyses

**Figure 7:**
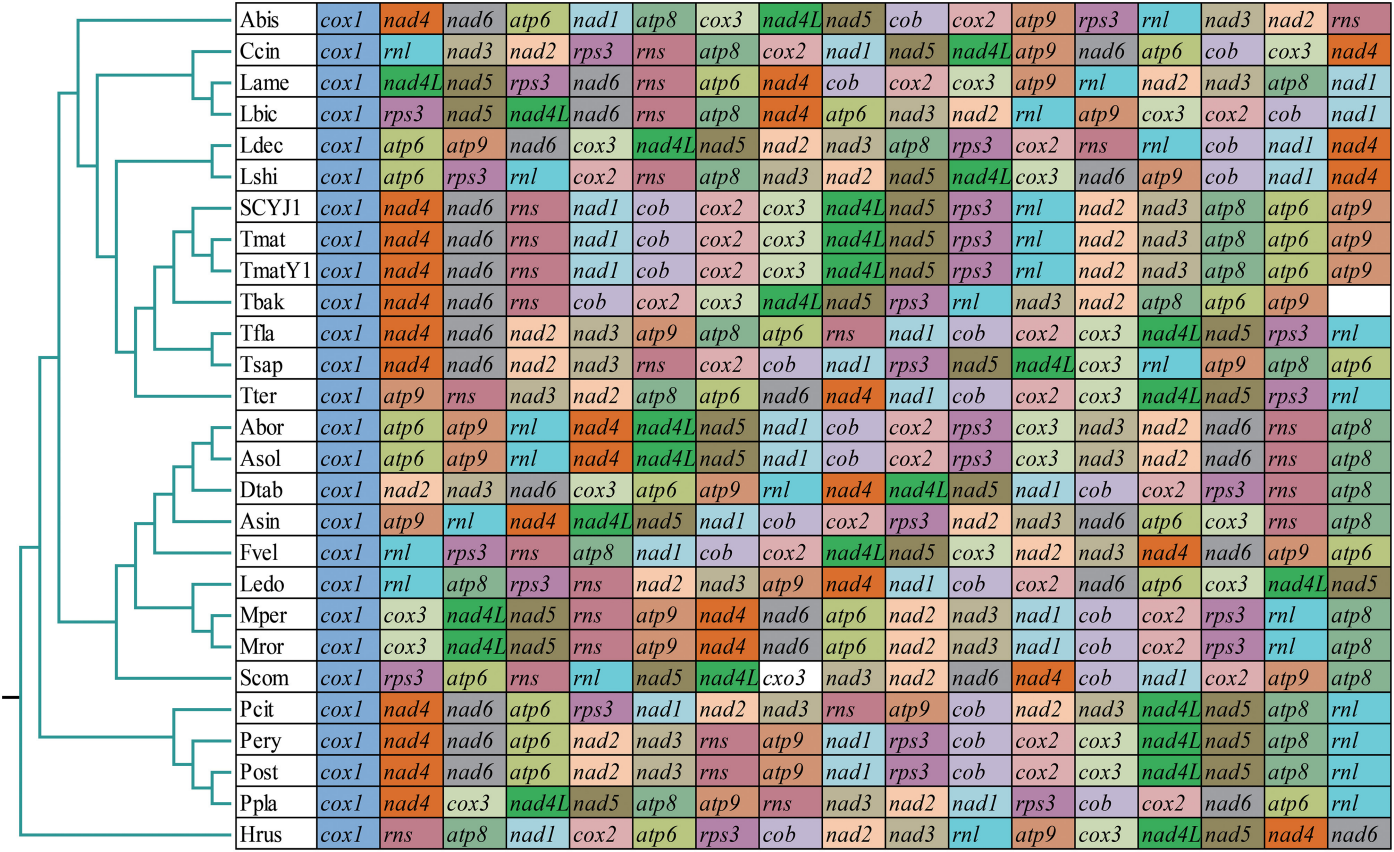
Gene order comparison between 27 *Agaricales* mitogenomes. Genes are represented with different-colored blocks. All genes are shown in order of occurrence in the mitochondrial genome, starting from *cox1*. Fourteen core protein-coding genes, one *rps3* gene, and two rRNA genes were included in the gene arrangement analysis. Species and NCBI accession number used for gene arrangement analysis in this study are listed in Supplementary Table 9.

The arrangement of mitochondrial genes could provide reference information for understanding the phylogenetic relationships between species (Sankoff et al., 1992). In this study, we found that the gene arrangement of the 27 *Agaricales* species varied significantly at the family level, indicating that large-scale gene rearrangements occurred in the evolution of *Agaricales* species (Figure 7). Identical gene arrangements were only observed between some species from the same genus, such as the *Moniliophthora* (Formighieri et al., 2008; Costa et al., 2012), *Pleurotus* (Li et al., 2018d), and *Armillaria* (Kolesnikova et al., 2019). In the genus *Tricholoma*, we found that the gene arrangement of the three *T. matsutake* species collected from different regions was identical, and large-scale gene rearrangements were detected between different species of *Tricholoma*, including gene transfer, insertion, deletion, and inversion events. In addition, we found that the *T. bakamatsutake* had a similar gene order with *T. matsutake* species, indicating that they were closely related, which was confirmed by phylogenetic analysis based on the combined mitochondrial gene set.

**Figure 8:**
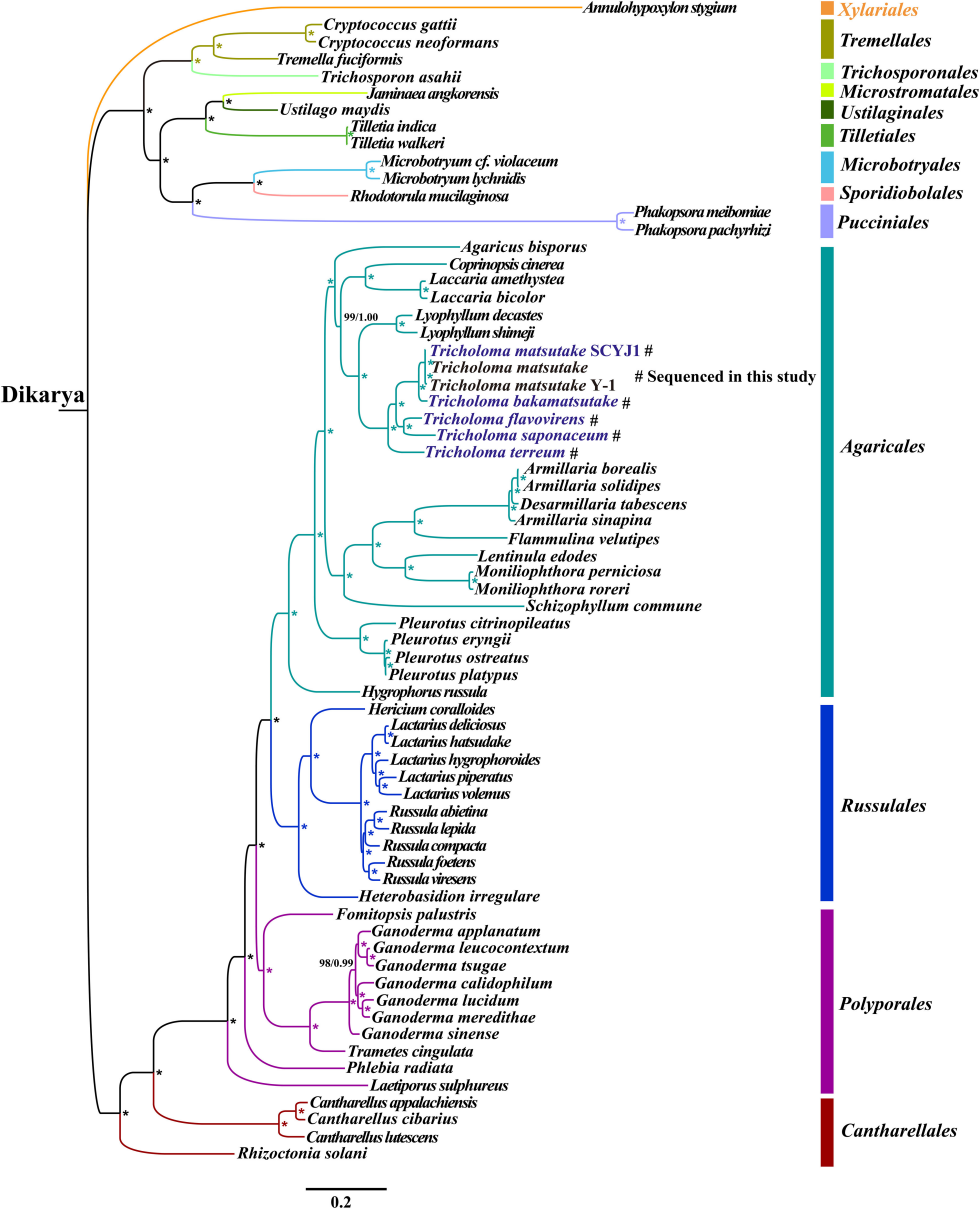
Molecular phylogeny of 67 *Basidiomycota* species based on Bayesian inference (BI) and maximum likelihood (ML) analysis of 15 protein-coding genes. Support values are Bayesian posterior probabilities (BPP; before slash) and bootstrap (BS) values. The asterisk indicates that the BS and BPP values on the evolutionary branch are 100 and 1.00, respectively. Species and NCBI accession numbers for genomes used in the phylogenetic analysis are provided in Supplementary Table 9.

Phylogenetic analysis using ML and Bayesian inference (BI) methods based on the combined mitochondrial gene set (14 core PCGs) yielded identical and well-supported tree topologies (Figure 8). All major clades within the trees were well-supported (BPP ≥ 0.99; BS ≥ 98). According to the phylogenetic tree, the 67 *Basidiomycota* species could be divided into 12 major clades, corresponding to the orders *Tremellales, Trichosporonales, Microstromatales, Ustilaginales, Tilletiales, Microbotryales, Sporidiobolales, Pucciniales, Agaricales, Russulales, Polyporales*, and *Cantharellales*. The 27 *Agaricales* species could be divided into four groups, wherein the first comprised only one species forming the *Hygrophorus* genus (Li et al., 2019d), and the second group comprised four species within the *Pleurotus* genus (Li et al., 2018d). The phylogenetic analyses indicated that *T. matsutake* was a sister species to *T. bakamatsutake*, and *T. flavovirens* was a sister species to *T. saponaceum*. The analyses also indicated that the *Tricholoma* genus showed close relationships with the *Lyophyllum* genus (Li et al., 2019e).

## Discussion

### Size Variations of Mitogenomes in *Tricholoma* Species

The mitogenome of fungi vary greatly in size, possibly caused by the accumulation of repeat sequences, transferred genes, and intron variations (Li et al., 2018e,f). In the present study, we found that the mitogenome size of *Tricholoma* species also varied greatly, and the largest *Tricholoma* mitogenome was 2.08 greater than the smallest mitogenome. We found that *T. bakamatsutake* with the largest mitogenome among *Tricholoma* species contained 22 introns, while *T. flavovirens*, containing the smallest mitogenome of *Tricholoma*, had only one intron. The results indicated that the dynamic changes of introns were one of the main factors contributing to the size variations of the mitogenomes in *Tricholoma*. Intergenic regions also played an important role in the size variation of *Tricholoma* mitogenomes. In addition, within the *T. matsutake* species, we found that the length of *T. matsutake* collected from Sichuan was 1,224 and 1,172 bp smaller than that from Japan (LC385608) and Korea (Yoon et al., 2016), respectively. Comparative mitogenomic analysis indicated that the *T. matsutake* collected from Sichuan has lost intron P9 (1,290 bp) in the *cox1* gene compared with that from Korea and Japan, indicating that the loss of the intron contributed to the contraction of the mitogenome in *T. matsutake* from Sichuan compared with that from Korea and Japan.

### Mitochondrial Content Evolution of *Tricholoma*

The natural gene transfer between nuclear and mitochondrial genomes plays an important role in the evolution of species (Adams and Palmer, 2003; Baris et al., 2017). Some of the mitochondrial genes were transferred to the nuclear genome, while some were retained (Adams and Palmer, 2003; Allen, 2015). In the present study, we observed several aligned fragments between nuclear and mitochondrial genomes of the five *Tricholoma* species, respectively, indicating that gene transfer may have occurred in *Tricholoma* during evolution. In addition, we found that the core PCGs in *Tricholoma* species varied in length and base composition, and the 14 core PCGs for energy metabolism were subjected to purifying selection. Interestingly, the *rps3* gene, a conserved gene in *Tricholoma* involved in the translation of the mRNA, demonstrated positive selection or relaxed selection between some *Tricholoma* species (Bullerwell et al., 2000). The *rps3* gene is an ancient gene in fungi, which evolved within the endosymbiotic model and presents varied evolutionary routes (Korovesi et al., 2018). The *rps3* gene was frequently observed to experience positive selection or relaxed selection in fungi (Wang et al., 2020b,c), and the selection pressure on *rps3* gene needs to be further verified. Most mitochondrial genes have been transferred to the nuclear genome during the evolution of eukaryotic lineages, which is considered to have many advantages (Bjorkholm et al., 2015). However, in the present study, we found some non-conserved PCGs in the *Tricholoma* species, most of which had unknown functions (Allen, 2015; Bjorkholm et al., 2015). Some non-conserved PCGs in the *Tricholoma* species encoded DNA polymerases, which are likely to be derived from mitochondrial plasmids (Wu et al., 2021). The results indicated that there are still some unknown functional proteins in *Tricholoma* species to be revealed, which would promote the understanding of evolution and function of fungal mitogenomes.

### Dynamics of Introns in *Agaricales*

The variation of introns was one of the main factors contributing to the size variation of the mitogenome in *Agaricales* (Hamari et al., 2002). In the present study, the characterization and dynamic changes of introns in the mitogenome of *Agaricales* were analyzed. Comparative intron analysis showed that the number of introns in the mitochondrial core PCGs of *Agaricales* varied greatly, ranging from 0 to 46, while most of these introns were located in *cox1* and *cob* genes, which may be due to the diverse insertion sites of the two genes. Introns could be divided into different Pcls according to their precise insertion site in the protein-coding region (Ferandon et al., 2013). Introns belonging to the same Pcl were considered to be orthologous (Ferandon et al., 2010). In the present study, we found that the Pcl of *Agaricales* introns varied greatly. Some introns were widely distributed in *Agaricales*, such as the P10 and P22 in the *cox1* gene and P19 in the *cob* gene. These introns may be inherited from the ancestors of *Agaricales*. However, some rare introns were only found in one of the 27 *Agaricales* species. Introns with the same insert sites were detected in distant species from other taxa (Himmelstrand et al., 2014), indicating that the potential transfer of introns or differential retention and loss occurred in the evolution of *Agaricales*. This phenomenon may also be due to the convergence of intron insertion sites in distant species. In addition, we also found the loss/gain of introns in the evolution of *Tricholoma* species. Compared with *T. matsutake* from Korea and Japan, *T. matsutake* from China lost the P9 intron of the *cox1* gene, indicating that even within the species of *T. matsutake*, the intron also varied in type or quantity. The impact of this phenomenon on the ecological adaptation and species differentiation of *T. matsutake* needs to be further analyzed.

### Gene Rearrangements and Phylogenetic Analysis

The arrangement of mitochondrial genes can provide important reference information for revealing the phylogeny and evolutionary status of eukaryotes (Sankoff et al., 1992). Reports indicated that the arrangement of mitogenome in fungi varied greatly, and the variation frequency was significantly higher than that of animals (Aguileta et al., 2014). Mitochondrial gene rearrangements in animals have been widely studied, and several models have been proposed to reveal the mechanism of mitochondrial rearrangements, including the tandem duplication-random loss (TDLR) (Xia et al., 2016) and duplication and nonrandom loss model (Lavrov et al., 2002). However, mitochondrial gene rearrangements in fungi have not been fully studied. In this study, we found that the mitochondrial gene arrangement varied greatly in *Agaricales*, even between closely related species. Large-scale gene rearrangements between *Tricholoma* species were detected, including mitochondrial gene transfer, insertion, deletion, and inversion events. The gene arrangement of the three *T. matsutake* species was consistent. *T. bakamatsutake* was found containing similar gene arrangement with *T. matsutake*, indicating a close phylogenetic relationship between the two species.

Mitochondrial genes have been widely used to study the phylogenetic relationships of eukaryotes, because of their single parent inheritance and rapid evolution rate (Beaudet et al., 2013; Andersen and Balding, 2018). However, due to the insufficiency of complete mitogenomes of fungi, the study on phylogenetic relationships of Basidiomycetes based on mitochondrial genomes was limited. So far, only one complete mitogenome from the *Tricholoma* genus has been published (Yoon et al., 2016), which limits the understanding of the evolution and phylogeny of *Tricholoma* species. The rapid development of the next generation sequencing technology promoted the acquisition of the mitogenome. As a supplement of the rDNA ITS, *RPB2*, and *EF1*α (Heilmann-Clausen et al., 2017; Reschke et al., 2018), mitochondrial gene molecular markers provide more abundant genetic information for reconstructing the phylogenetic tree of fungi or analyzing the evolution of fungi (Li et al., 2021). In addition, many Basidiomycete species have limited morphological features for recognition, and some morphological features are easy to overlap, leading to the confusion of Basidiomycetes classification. Phylogenetic analysis based on mitochondrial genes effectively promotes the classification and species identification of Basidiomycetes. In this study, we obtained identical and well-supported tree topologies of Basidiomycota based on the combined mitochondrial gene set using both ML and BI methods. *Tricholoma* species have a relatively close phylogenetic relationship with *Lyophyllum* species (Li et al., 2019e), which was consistent with previous phylogenetic results based on nuclear gene markers (Moncalvo et al., 2000). The results showed that the mitogenome was an effective molecular marker to analyze the phylogenetic relationships of Basidiomycetes. More Basidiomycetes mitogenomes are needed to reveal the phylogenetic relationships of all Basidiomycetes.

## Conclusion

In this study, five mitogenomes from the *Tricholoma* genus were sequenced, assembled, and compared. Comparative mitogenomic analyses indicated that intron was one of the main factors contributing to size variations of *Tricholoma* mitogenomes, even within species. The introns of *Agaricales* mitogenomes experienced frequent loss/gain events. *T. matsutake* collected from Sichuan had lost one intron P9 (1,290 bp) in the *cox1* gene compared with that from Korea and Japan, contributing to the contraction of the mitogenome in *T. matsutake* from Sichuan. In addition, large aligned fragments were detected between respective *Tricholoma* mitogenomes and their nuclear genomes, indicating that potential gene transfers may have occurred in the evolution of *Tricholoma* species. Evolutionary analysis showed that the core PCGs for energy metabolism were subject to a purifying selection, while the *rps3* gene was subject to positive selection or relaxed selection. In addition, large-scale gene rearrangements were detected between some *Tricholoma* species and other related *Agaricales* species. Phylogenetic analysis using the BI and ML methods based on a combined mitochondrial gene set yielded identical and well-supported tree topologies, and the *Tricholoma* genus showed close relationships with the *Lyophyllum* genus.This study promoted the understanding of the genetics, evolution, and phylogeny of the *Tricholoma* genus and related species.

## Data Availability Statement

Publicly available datasets were analyzed in this study. This data can be found at: The five *Tricholoma* mitogenomes, including *T. matsutake* SCYJ1, *T. bakamatsutake, T. terreum, T. flavovirens*, and *T. saponaceum*, were submitted to GenBank under accession numbers MN873034, MN873035, MN873036, MN873037, and MN873038, respectively.

## Author Contributions

QL and WH: conceived and designed experiments. PL, CX, HF, WT, and XJ: performed the experiments and analyze the data. QL and XW: wrote and revise the paper. All authors contributed to the article and approved the submitted version.

## Conflict of Interest

The authors declare that the research was conducted in the absence of any commercial or financial relationships that could be construed as a potential conflict of interest.
